# Updated RDP taxonomy and RDP Classifier for more accurate taxonomic classification

**DOI:** 10.1128/mra.01063-23

**Published:** 2024-03-04

**Authors:** Qiong Wang, James R. Cole

**Affiliations:** 1 Health & Biosciences, International Flavors & Fragrances, Inc., Wilmington, Delaware, USA; 2 Department of Plant, Soil and Microbial Sciences, Center for Microbial Ecology, Michigan State University, East Lansing, Michigan, USA; University of Strathclyde, United Kingdom

**Keywords:** 16S rRNA, taxonomy, taxonomic classification, microbiome

## Abstract

The RDP Classifier is one of the most popular machine learning approaches for taxonomic classification due to its robustness and relatively high accuracy. Both the RDP taxonomy and RDP Classifier have been updated to incorporate newly described taxa and recent changes to prokaryotic nomenclature.

## ANNOUNCEMENT

The RDP taxonomy is one of the few widely accepted prokaryotic taxonomies used for bacteria and archaea identification and has been suggested as the preferred training set for several rRNA-based taxonomic classifiers (1). This taxonomy, with a few exceptions, is based on validly named species and higher taxonomic ranks, using rRNA gene sequences from species type strains. The RDP Classifier is a software tool that assigns rRNA sequence data from bacterial and archaeal 16S rRNA genes and fungal LSU and ITS genes to the corresponding taxonomy models (2–4). The RDP Classifier is particularly valuable in microbiome studies and remains highly cited even 16 years after its initial publication, with 2,055 citations in 2022 alone (Google Scholar, 27 September 2023).

**Fig 1:**
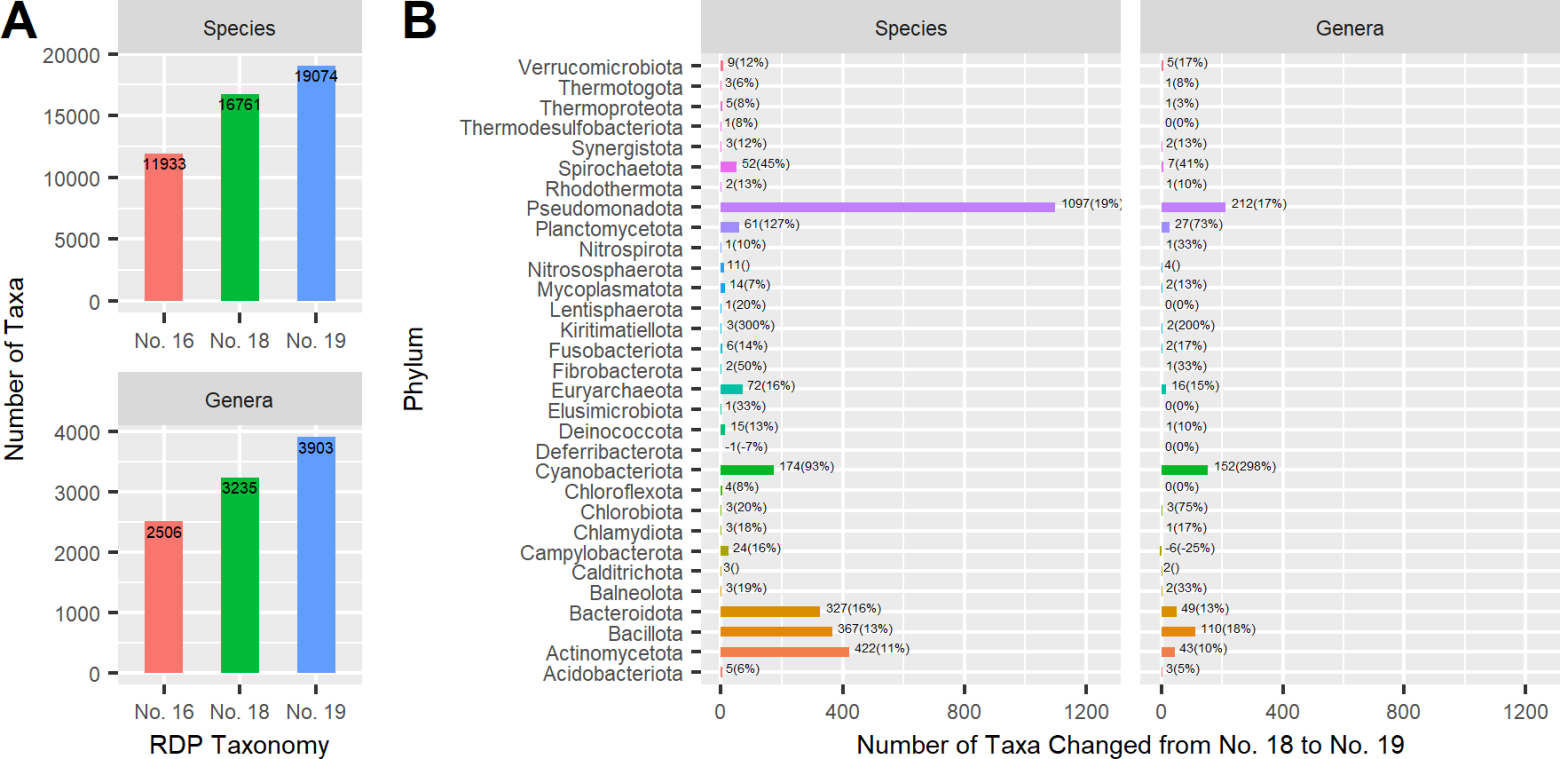
Taxa increases in training set No. 19 compared to previous releases. (**A**) Taxa at species and genus rank from three most recent RDP taxonomy training sets: 16 (2016), 18 (2020), and 19 (2023). (**B**) Species and genera increase by phylum in No. 19 compared to No. 18 (using the corresponding No. 19 phylum names). Percentages are in parentheses. Only phyla with changes are shown.

We have updated the RDP taxonomy to training set No. 19, released in 2023, to incorporate newly described taxa and recent major changes in the nomenclature of prokaryotes. This update consists of 24,642 sequences from 19,074 species and 3,903 genera, an increase of 2,313 (13.8%) and 668 (20.6%), respectively, compared to No. 18 (Fig. 1A). Phylum *Pseudomonadota* has the highest number of changes (Fig. 1B). In addition to the inclusion of new species, this release contains thousands of taxonomic changes and name revisions, many based on the inclusion of newly valid phylum names (5) and regularization of name at other ranks. Future releases will include stable SeqCode identifiers and associated taxonomy for well-studied uncultivated microorganisms (6). We tested the training set for internal consistency using exhaustive leave-one-sequence-out testing. The classification accuracies are 99.9%, 99.8%, 99.7%, 99.1%, and 97.2% for near-full-length and 99.7%, 99.4%, 98.4%, 96.0%, and 86.4% for 250-bp length fragments at phylum, class, order, family, and genus ranks, respectively.

The RDP taxonomy was updated primarily using the International Journal of Systematic and Evolutionary Microbiology through Volume 73, Issue 5, 2023, the List of Prokaryotic names with Standing in Nomenclature (7), and public sequence repositories. The curation process was guided by the phylogenetic trees from the All-Species Living Tree Project (LTP_06_2022) (8).

The RDP Classifier has also been updated to release 2.14 with numerous enhancements and small bug fixes that were applied over time (9). A notable feature is the cross-validation testing, which is particularly useful for users training the Classifier using their own data and taxonomy. It reports classification accuracy rate and identifies the misclassified sequences that help detect the errors in the taxonomy. Another enhancement is the option to adjust the assignment counts of the taxa based on the 16S gene copy number information from release 5.6 of the ribosomal RNA operon copy number database (10).

## Data Availability

RDP taxonomy training set No. 19, in both RDP Classifier and Qiime2 format, along with RDP Classifier release 2.14, are available on Zenodo and on SourceForge. An additional file containing taxonomy information to the species level is included in this release for users who wish to differentiate closely related species. Instructions for installing the RDP Classifier and tutorials on its use can be found at GitHub.
